# Preconception care: advancing from ‘important to do and can be done’ to ‘is being done and is making a difference’

**DOI:** 10.1186/1742-4755-11-S3-S8

**Published:** 2014-09-26

**Authors:** Elizabeth Mason, Venkatraman Chandra-Mouli, Valentina Baltag, Charlotte Christiansen, Zohra S Lassi, Zulfiqar A Bhutta

**Affiliations:** 1 Department of Maternal Newborn Child and Adolescent Health, World Health Organization; 2 Department of Reproductive Health and Research, World Health Organization; 3 Division of Women and Child Health, Aga Khan University Karachi, Pakistan

**Keywords:** Preconception, cost, call for action

## Abstract

**There is a growing evidence base for preconception care - – the provision of biomedical, behavioral and social interventions to women and couples before conception occurs.** Firstly, there is evidence that health problems, problem behaviours and individual and environmental risks contribute to poor maternal and child health outcomes. Secondly, there are biomedical, behavioural and social interventions that when delivered before conception occurs, effectively address many of these health problems, problem behaviours and risk factors. And thirdly, there is emerging experience of how to deliver these interventions in low and middle income countries (LMIC).

**The preconception care interventions delivered and whom they are delivered to, will need to be tailored to local realities.** The package of preconception care interventions delivered in a particular setting will depend on the local epidemiology, the interventions already being delivered, and the resources in place to deliver additional interventions. Although a range of population groups could benefit from preconception care, prioritization based on need and feasibility will be needed.

**There are both potential benefits and risks associated with preconception care.** Preconception care could result in large health and social benefits in LMIC. It could also be misused to limit the autonomy of women and reinforce the notion that the focus of all efforts to improve the health of girls and women should be at improving maternal and child health outcomes rather than at improving the health of girls and women as individuals in their own right.

**There are challenges in delivering preconception care.** While the potential benefits of preconception care programmes could be substantial, extending the traditional Maternal and Child Health package will be both a logistic and financial challenge.

**We need to help countries set and achieve pragmatic and meaningful short term goals.** While our long-term goal for preconception care should be for a full package of health and social interventions to be delivered to all women and couples of reproductive age everywhere, our short-term goals must be pragmatic. This is because countries that need preconception care most are the ones least likely to be able to afford them and deliver them. If we want these countries to take on the additional challenge of providing preconception care while they struggle to increase the coverage of prenatal care, skilled care at birth etc., we must help them identify and deliver a small number of effective interventions based on epidemiology and feasibility.

## Introduction

The world has made significant improvement in saving the lives of mothers and children since Millennium Development Goals 4 and 5 were adopted by world leaders in 2000. Yet even now, with existing effective interventions and public health commitment greater than ever before, there are still 287000 maternal and 2.9 million newborn deaths each year, with an additional 2.6 million stillbirths [1,2] Further, there are growing problems such as obesity and other chronic health problems with their attendant maternal and fetal risks. [3] A significant proportion of newborn deaths are due to prematurity, which may result from a number of factors. [4] Most disconcertingly, 90% or more of maternal and child mortality remains concentrated in South Asia and Sub-Saharan Africa, countries that simply lack the resources to deliver existing, effective interventions that could prevent many of these deaths ([5]. Moving towards preconception care offers the potential for earlier risk assessment and intervention that can benefit the woman or couple even before pregnancy and ensure the healthiest possible start for the newborn child.

This paper is the last in a set of eight papers on preconception care which define the field [6], outline the evidence in a number of areas of public health importance [7-11] and describes packages that could be delivered in different contexts [12]

## What is pre-conception care?

**Figure 1:**
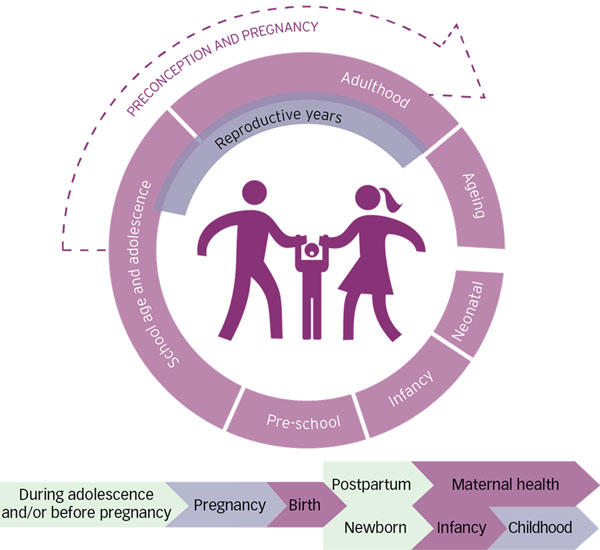


Preconception care is the provision of biomedical, behavioral and social interventions to women and couples before conception occurs to address health problems, behaviors that could lead to health problems, and individual or environmental risk factors that could contribute to maternal or childhood mortality and morbidity. Its ultimate aim is improved maternal and child health outcomes [6]. Preconception care covers the period before a first pregnancy occurs, and between two pregnancies. This is illustrated in Figure 1.

All babies and children have the right to survive, and to grow and develop in good health. Similarly, all women and men have the right to be healthy – physically, psychologically and socially. For this, strong public health programmes that use a life course perspective are needed.

In low and middle income countries (LMIC), such programmes are weak or nonexistent. Even where they exist, they do not guarantee that women enter pregnancy in good health. That is why in all high income countries (as well as in LMIC) prenatal care programmes are in place to promote good health, prevent health problems and respond to them if they occur during pregnancy [13].

To achieve MDGs 4 and 5 targets, heroic effects are underway in many LMIC to improve access to a package of maternal and child health (MCH) interventions [14]. Although progress is being made, substantial numbers of pregnant women in many LMICs, especially in sub Saharan Africa and South Asia, are not going to achieve MDGs 4 and 5 targets [15], and do not receive the minimum four prenatal care consultations recommended by the World Health Organization (WHO).

Even in countries where the majority of women receive these consultations, care in the antenatal period may be too late to address important health problems such as obesity, problem behaviours such as tobacco use or risk factors such as exposure to domestic violence. Extending the existing MNCH continuum with one step before prenatal care, expanding it to include interventions that are not currently part of the package, and actively including men and boys will be challenging to do but will improve the health of girls and boys, men and women before pregnancy occurs.

## Evidence

The evidence has been well elucidated in the previous papers [7-11]. Firstly, there is convincing evidence that health problems e.g. nutritional problems such as anaemia and obesity, vaccine preventable diseases such as rubella, and mental health problems such as depression, contribute to poor maternal and child health outcomes. There is also convincing evidence that problem behaviours e.g. tobacco and alcohol use, and risk factors e.g. individual genetic conditions such as thalassemia, and environmental health risks e.g. exposure to chemicals and radiation can also contribute to poor maternal and child health outcomes as do too early and rapid, successive pregnancies.

Secondly, there are effective biomedical, behavioural and social interventions that when delivered before conception occurs, address many health problems, problem behaviours and risk factors [7-10,13,16,17]. For example, rubella vaccination can prevent congenital rubella and folic acid supplementation can prevent neural tube defects. In other areas, there is less evidence of effective interventions. For example, the effectiveness of interventions in preventing child marriage in poor and traditional communities, or in preventing intimate partner violence, is less straightforward.

Thirdly, there is limited evidence of effective ways of delivering these interventions in LMIC – especially the social and behavioural interventions. Through an extensive consultative process, research priorities for preconception care have been identified [18]. The research priority-setting exercise focused on the development and delivery of existing interventions during the preconception period in LMICs. The research questions that received the highest scores highlighted the need to develop strategies to increase coverage of basic interventions such as improving nutrition; reproductive planning for adolescents; contraception; prevention, detection and treatment of chronic conditions that affect maternal health; immunization, diagnosis and treatment of infectious diseases; and reducing harmful environmental smoke exposure. The highest priorities also included a systems-based approach to increase preconception care services in LMICs including integration with other programmes; task-shifting to Community Health Workers; improving supply chains for preconception care commodities; partnerships with media and information technology; maximizing demand for and uptake of preconception interventions, especially by adolescents.

## Potential benefits of preconception care

Preconception care can contribute to reducing maternal and childhood mortality and morbidity in both high and low income countries. In the former, in addition to optimizing general preconception health and risk awareness of the population as a whole, it can address the relatively high levels of maternal and childhood mortality and morbidity in pockets of socially marginalized and economically deprived families and communities.

Preconception care could result in large health and social benefits in LMIC. It could make a substantial contribution to reducing maternal and childhood mortality and morbidity and to the health of babies as children as they grow into adolescence and adulthood. Further, by supporting women to make well-informed and well-considered decisions about their fertility and health, preconception care could contribute to social and economic development of families and communities. By creating awareness of the importance of men’s health and men’s behaviour on maternal and child health outcomes, and by promoting male involvement, it could result in additional benefits. Finally, it could also contribute to strengthening the wider public health agenda by linking public health programmes, many of which are currently weakly linked at best

## What interventions to deliver, how to deliver them to, and whom to target

As discussed above, there is a menu of effective interventions to address health problems, problem behaviours and risk factors in the preconception period which increase the likelihood of maternal and childhood mortality and morbidity (Table 1) [14].

**Table 1:** Evidence-based preconception care interventions to address health problems contributing to maternal and child mortality

Problems that can be addressed by preconception care interventions	Examples of evidence-based interventions
Nutritional deficiencies and disorders	• Screening for anaemia and diabetes • Supplementing iron and folic acid • Information, education and counselling • Monitoring nutritional status • Supplementing energy- and nutrient-dense food • Management of diabetes, including counselling people with diabetes mellitus • Promoting exercise • Iodization of salt

Vaccine-preventable infections	• Vaccination against rubella • Vaccination against tetanus and diphtheria • Vaccination against Hepatitis B

Tobacco use	• Screening of women and girls for tobacco use (smoking and smokeless tobacco) at all clinical visits using “5 As” (ask, advise, assess, assist, arrange) • Providing brief tobacco cessation advice, pharmacotherapy (including nicotine replacement therapy, if available) and intensive behavioural counselling services • Screening of all non-smokers (men and women) and advising about harm of second-hand smoke and harmful effects on pregnant women and unborn children

Environmental risks	• Providing guidance and information on environmental hazards and prevention • Protecting from unnecessary radiation exposure in occupational, environmental and medical settings • Avoiding unnecessary pesticide use/providing alternatives to pesticides • Protecting from lead exposure • Informing women of childbearing age about levels of methyl mercury in fish • Promoting use of improved stoves and cleaner liquid/gaseous fuels

Genetic disorders	• Taking a thorough family history to identify risk factors for genetic conditions • Family planning • Genetic counselling • Carrier screening and testing • Appropriate treatment of genetic conditions • Community-wide or national screening among populations at high risk

Early pregnancies, unwanted pregnancies, and rapid successive pregnancies	• Keeping girls in school • Influencing cultural norms that support early marriage and coerced sex • Providing age-appropriate comprehensive sexuality education • Providing contraceptives and building community support for preventing early pregnancy and contraceptive provision to adolescents • Empowering girls to resist coerced sex • Engaging men and boys to critically assess norms and practices regarding gender-based violence and coerced sex • Educating women and couples about the dangers to the baby and mother of short birth intervals

Sexually transmitted infections	• Providing age-appropriate comprehensive sexuality education and services • Promoting safe sex practices through individual, group and community-level behavioural interventions • Promoting condom use for dual protection against STIs and unwanted pregnancies • Ensuring increased access to condoms • Screening for STIs • Increasing access to treatment and other relevant health services

HIV	• Family planning • Promoting safe sex practices and dual method for birth control (with condoms) and STI control • Provider-initiated HIV counselling and testing, including male partner testing • Providing antiretroviral therapy for prevention and pre-exposure prophylaxis • Providing male circumcision • Providing antiretroviral prophylaxis for women not eligible for, or not on, antiretroviral therapy to prevent mother-to-child transmission • Determining eligibility for lifelong antiretroviral therapy

Infertility and subfertility	• Creating awareness and understanding of fertility and infertility and their preventable and unpreventable causes • Defusing stigmatization of infertility and assumption of fate • Screening and diagnosis of couples following 6–12 months of attempting pregnancy, and management of underlying causes of infertility/sub-fertility, including past STIs • Counselling for individuals/couples diagnosed with unpreventable causes of infertility/sub-fertility

Female genital mutilation	• Discussing and discouraging the practice with the girl and her parents and/or partner • Screening women and girls for FGM to detect complications • Informing women and couples about complications of FGM and about access to treatment • Carrying out defibulation of infibulated or sealed girls and women before or early in pregnancy • Removing cysts and treating other complications

Mental health disorders	• Providing educational and psychosocial counselling before and during pregnancy • Counselling and treating depression in women planning pregnancy and other women of childbearing age • Strengthening community networks and promoting women’s empowerment • Improving access to education for women of childbearing age • Reducing economic insecurity of women of childbearing age

Psychoactive substance use	• Screening for substance use • Providing brief interventions and treatment when needed • Treating substance use disorders, including pharmacological and psychological interventions • Providing family planning assistance for families with substance use disorders (including postpartum and between pregnancies) • Establishing prevention programmes to reduce substance use in adolescents

Interpersonal violence	• Health promotion to prevent dating violence • Providing age-appropriate comprehensive sexuality education that addresses gender equality, human rights, and sexual relations • Combining and linking economic empowerment, gender equality and community mobilization activities • Recognizing signs of violence against women • Providing health care services (including post-rape care), referral and psychosocial support to victims of violence • Changing individual and social norms regarding drinking, screening and counselling of people who are problem drinkers, and treating people who have alcohol use disorders

In order to achieve each outcome, a combination of interventions needs to be delivered using a combination of methods. Here are three illustrative examples:

• Preventing child marriage by empowering girls, enabling them to enroll in school and complete their secondary schooling, advocating with the families and communities, and formulating/implementing laws that prevent child marriage.

• Nutrition monitoring and education, and the provision of supplementary foods can reduce underweight. Education and monitoring can be done in health facilities and community settings. Social welfare programmes, non-governmental organizations or civil society bodies may provide food supplementation to needy individuals and families.

• Educating women and couples about the dangers of short birth intervals and promoting/providing contraceptives can reduce rapid, successive pregnancies. Education can be done in health facilities and community settings, and through mass media. Contraceptive provision can be done through a range of public and private outlets.

The preconception care interventions delivered in a particular setting will depend on the local epidemiology, the interventions already being delivered, and the mechanisms resources in place to deliver additional interventions. For example, following reviews of the national maternal health programme, the Ministry of Health of Sri Lanka realized that indirect causes resulting from pre-existing cardiac conditions and violence were contributing to a growing share of ’maternal mortality and serious morbidity. In response to Ministry of Health designed, piloted and implemented a programme to identify newly-weds and to reach them with information, screening and services. [19,20]

A logical target group for initiating work in this area is individuals and couples contemplating a pregnancy. As they are considering a pregnancy, they are likely to be receptive to inputs on what they could do to increase the likelihood of positive maternal and child health outcomes. Identifying and reaching them will be challenging. The next group to consider is individuals who are not currently contemplating a pregnancy. There are risks inherent in this, however: it assumes, incorrectly, that all men and women want to be parents. Further, individuals and couples seeking assistance for pregnancy prevention may not be ready to take on board advice on what they should consider if and when do contemplate having a child. In both groups – those who are and are not contemplating a pregnancy – both men and women should be targeted. Men’s health and men’s health behaviours have important implications for the health of their partners and their children, and men have important roles to play as husbands, partners, fathers and community members. Effective ways of doing this must be developed and employed widely. In both high and low income countries, a special effort should be made to target individuals, couples, families and communities who are socially and economically marginalized and so more vulnerable to health and social problems. Adolescent girls are especially vulnerable in many LMIC, and without special attention, their needs are likely to be neglected. Efforts to reach those who are marginalized and vulnerable must ensure that they do not lead to blaming and stigmatizing them. Finally, couples with previous adverse reproductive outcomes, and individuals with preexisting genetic risks, genetic conditions such as thalassemia or health conditions such as diabetes or epilepsy need to be reached with interventions tailored to their individual needs.

## Potential risks of preconception care

Attention needs to be given to ensure that preconception care is not misused to limit the autonomy of women and to undermine their rights. A strong focus on preconception care could run the risk of defining girls as being in a preconception state even before their menarche, and women as being in a preconception state for the entire duration of their fertile period when they are not pregnant. This could lead to women

• being barred from participating in situations and taking up work in some areas on the grounds that it would increase the risk of adverse maternal and child health outcomes;

• vilified or prosecuted for their conduct, such as smoking or drinking alcohol.

Further, an emphasis on preconception care could reinforce the notion that the focus of all efforts to improve the health of girls and women should be at improving maternal and child health outcome rather than at improving the health of girls and women as individuals in their own right. In addition, blanket approaches targeting the entire population of girls and women could be seen to imply that all girls and women will invariably become mothers.

Governments should be alert to these risks. They should put in place mechanisms to reduce their likelihood, to detect them when they appear and to respond promptly and effectively if they do. Civil society bodies such as experienced women’s rights organizations could play a valuable role both embedded in government-headed committees and working groups and in playing the role of independent watch dogs

## Challenges of delivering preconception care in LMIC

Extending the MCH package will be both a logistic and financial challenge. As a senior official from Thailand’s Ministry of Public Health said in a South East Asian expert group consultation on preconception care (New Delhi, India, August 2013): “A programmatic goal of screening, providing prophylactic medication and treating all pregnant women for anaemia is very different from a programmatic goal of ensuring that all women of reproductive age are anaemia free, is very different in scale, complexity and cost. I would not know where to begin”.

While our long-term goal for preconception care should be for a full package of health and social interventions to be delivered to all women and couples of reproductive age everywhere, our short-term goals must be pragmatic. This is because countries that need preconception care most are the ones least likely to be able to afford them and deliver them.

If we want these countries to take on the additional challenge of providing preconception care while they struggle to increase the coverage of prenatal care, skilled care at birth etc., we must help them identify and deliver a small number of effective interventions based on epidemiology and feasibility.

## What will it cost?

Evidence from high income countries suggests that preconception care programmes are effective and result in cost savings. One example is the prevention of complications resulting from diabetes in mothers and children. While delivering preconception care along with prenatal care requires additional resources, these costs are balanced by the savings from averted complications [21,22]. A study in the USA showed that that medical care before conception resulted in cost savings compared with prenatal care only [21]. In addition to achieving its intended health benefits, preconception care could can also substantially reduced costs. Another study in the USA that compared pregnancy outcomes, resource utilization and costs among women with diabetes who received and did not receive preconception care, concluded that net cost saving associated with preconception care was approximately $34,000 per patient [22]. Savings resulted from significantly less frequent hospitalization, shorter inpatient stays, significantly shorter length of stay after delivery, as well as lower intensity of care and shorter length of stay for infants of mothers who received preconception care. Not only were the savings substantial, they also occurred in the short term.

The cost effectiveness of a preconception programme designed specifically for teenagers was reported by yet another study in the USA [23]. A preconception education/counseling reproductive health programme specifically tailored for teenagers (READY-Girls) was shown to be cost-effective in a setting where 6.7% of girls 15–20 years of age have an unplanned pregnancy each year. With relatively low cost the programme resulted in positive cognitive, psychosocial, and behavioral outcomes. It was estimated that if the READY-Girls programme reduced the absolute risk of an unplanned pregnancy by 0.6% or the relative risk of an unplanned pregnancy by 10%, the programme would be cost-neutral. If the programme reduced the risk of unplanned pregnancy by more than this threshold, it would be cost-saving [23].

The studies suggest that for both health and economic reasons, clinical practice and health policy should support the provision of preconception care for women with diabetes. More research needs to be done on the effectiveness and cost of preconception care programmes in contexts other than diabetes, and in different social and cultural contexts.

Data on the effectiveness, the costs and the cost effectiveness of programmes in LMICs are not available. Reaching all girls and young women, and all boys with the entire package of interventions listed in table 1 will be both prohibitively expensive and extremely difficult to do. But as Sri Lanka has shown, using existing MCH staff and existing MCH systems and structures, the continuum of care can to extended to a selected target group (i.e. newly married couples) with a selected set of interventions (e.g. education and counselling to prevent domestic violence, and screening and treatment/referral for pre-existing cardiac and neurological conditions).”

## Monitoring and evaluating preconception programmes

The ultimate test of the effectiveness of preconception care programmes will be the reduction in the levels of maternal and child mortality and morbidity. Key indicators for each preconception programme area will need to be developed, and monitored to demonstrate the results of these programmes. The indicators will need to capture inputs, processes, outputs (quality and coverage), outcomes and impact. They will also need to draw on existing globally agreed monitoring frameworks, such as for the global Non-Communicable Diseases (NCDs) monitoring framework [25] and the monitoring framework of the Commission on Information and Accountability (CoIA) of the *Every Woman*, *Every Child* initiative [15].

## Call to action

The acceptance, application and full realization of the benefits – both in terms of health outcomes and cost savings – of preconception care programmes both nationally and internationally, will depend on the contributions of many individuals, groups and institutions. Local, national, regional and global actions are necessary to improve the availability of and access to preconception care interventions.

**Figure 2:**
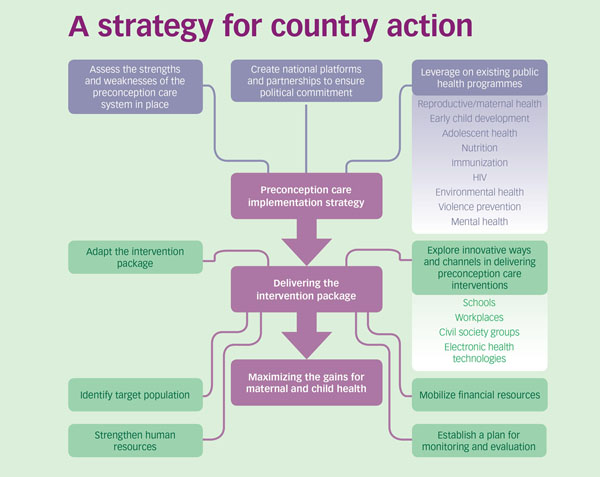


At the national level, governments will need to adopt an informed national strategy to put in place sustainable preconception care programmes (Figure 2).

Countries are at different stages in their current application of preconception care interventions. They also face different socio-cultural and economic contexts in which preconception care programmes are happening or will happen. In regions and countries where adolescent pregnancy rates are high, and early marriage common, preconception care will overlap with adolescent health programmes and projects. For example, in South East Asian expert group consultation on preconception care (New Delhi, India, August 2013) it was agreed that pre-conception care be introduced in two streams – one with a primary focus on reducing maternal and newborn mortality and morbidity, and the other with a focus on enabling older children and adolescents to make healthy transitions into adulthood.

While there are differences between and within countries, there are also similarities. Global and regional platforms for experience and expertise sharing may therefore be a useful way to accelerate knowledge transfer and mutual support between countries. WHO and international partners may play an important role in facilitating such platforms.

Ministries of Health will need support that goes beyond information exchange. They will need support to assess the situation, to short list priority interventions, to identify whom to deliver them to, to define how to deliver them, and to consider what it will take and cost to do so. And they will need support to translate this vision into a realistic plan and budget, and support to finance, implement and monitor implementation.

WHO is also committed to support countries to:

• Create regional/national platforms and partnerships to advance preconception care interventions.

• Introduce professionals in countries to international experience, research, evidence and good practices.

• Analyze and understand the strengths and weaknesses of the preconception care system in place, and opportunities for improvement.

• Explore various delivery strategies for preconception care interventions, and their comparative advantages in terms of coverage, feasibility, acceptability and cost.

• Adapt the package of preconception care interventions to regional and country priorities, and health systems contexts.

• Explore and document innovative ways to deliver preconception care outside the traditional maternal and child health programmes, while recognizing the importance of integrated delivery mechanisms.

• Monitor, evaluate and document progress. [12,13]

Leveraging on existing alliances will accelerate success. For example, the global alliance that has been forged to support countries in achieving the MDG 4 and 5 targets can play an important role in engaging policy makers and planners in dialogue, in stimulating and supporting learning by doing, and in documenting, evaluating and sharing country experiences. Partnering with the NCD community is very important, given the high profile of the NCDs in global health agenda and governments’ commitment to address NCDs within an explicit monitoring framework. The “Draft action plan for the prevention and control of noncommunicable diseases 2013–2020” which was discussed at the 66th World Health Assembly in May 2013, recognizes preconception care, as part of the national policy framework, as an important contributor to noncommunicable disease prevention and control [25].

We have achieved a global consensus that preconception care is important to do and can be done. We must now set our sights on the objective: Preconception care is being done, is being done *well*, and is making a difference.

## Competing interests

The authors declare that they have no competing interests.

## Additional files

In line with the journal's open peer review policy, copies of the reviewer reports for this article are included in Additional file 1.

## Supplementary Material

Additional file 1 Peer review files. Click here for file

## Declarations

This article has been published as part of *Reproductive Health* Volume 11 Supplement 2, 2014: Preconception interventions. The full contents of the supplement are available online at http://www.reproductive-health-journal.com/supplements/11/S3. Publication charges for this collection were funded by the Partnership for Maternal, Newborn & Child Health (PMNCH).
