# Supplementary material for: Preconception care: advancing from ‘important to do and can be done’ to ‘is being done and is making a difference’
Source: Reprod Health. 2014 Sep 26;11(Suppl 3):S8. doi: 10.1186/1742-4755-11-S3-S8 (PMC4196570; doi:10.1186/1742-4755-11-S3-S8)
Supplement: Additional file 1 — Peer review files. [file 1742-4755-11-S3-S8-S1.pdf]

Referee's comments to the authors– this sheet <u>WILL</u> be seen by the author(s) and published with the article

Title	Preconception care: Advancing from 'important to do and can be done' to 'is being done and is making a difference'
Author(s)	Elizabeth Mason*, Venkatraman Chandra-Mouli, Valentina Baltag, Charlotte Christiansen, Zohra S Lassi, Zulfiqar A Bhutta
Referee's name	Susan Sawyer

When assessing the work, please consider the following points, where applicable:

1. Is the question posed by the authors new and well defined?
2. Are the methods appropriate and well described, and are sufficient details provided to replicate the work?
3. Are the data sound and well controlled?
4. Does the manuscript adhere to the relevant standards for reporting and data deposition?
5. Are the discussion and conclusions well balanced and adequately supported by the data?
6. Do the title and abstract accurately convey what has been found?
7. Is the writing acceptable?

Please make your report as constructive and detailed as possible in your comments so that authors have the opportunity to overcome any serious deficiencies that you find and please also divide your comments into the following categories:

- Major Compulsory Revisions (which the author must respond to before a decision on publication can be reached)
- Minor Essential Revisions (such as missing labels on figures, or the wrong use of a term, which the author can be trusted to correct)
- Discretionary Revisions (which are recommendations for improvement but which the author can choose to ignore)

Where possible please supply references to substantiate your comments.

When referring to the manuscript please provide specific page and paragraph citations where appropriate.

General comments:

This paper is a 'call to arms' about the value of preconception care. The lead author is in a prominent position in Maternal, Newborn Child and Adolescent Health at WHO. The senior author is a highly regarded senior academic from Pakistan and Canada. The authors argue that in the context of 'growing evidence' for pre-conception care, effective interventions to address health issues and emerging experience in delivering these interventions in LMIC, that the time is now right to translate such a vision to reality. This is a grand vision. The authors describe a set of issues that need to be addressed before such a vision could be implemented. The paper is generally well written and logically argued, and the figure and table provide nice summary information.

The major difficulty in reviewing this paper is that, as the reviewer, I do not have access to the quality of evidence that the paper refers to. Having independently reviewed one of the other papers within the series, makes me question the quality of evidence on which the argument is based. In order to do justice to the review, it feels like one would need access to the series of systematic reviews that are referred to.

(continue on the next sheet)

Continued: I will address my major comments under each heading.

What is pre-conception care?

According to the definitions presented, pre-conception care *could* constitute any care or intervention prior to pregnancy that aims to improve the health of both women and men, and through doing, would expect to improve the health of future children. This is a laudable goal, albeit one that feels somewhat unrealistic when articulated within the preconception frame.

One major concern about the paper in its current form is that it fails to critically question the different periods that could be targeted for pre-conception interventions, in terms of effectiveness, feasibility, or cost. As I see it, this could be one of the most important contributions the paper could make. A diagram would be of particular value here in articulating the potential periods of interest within the lifecourse.

In particular, Figure 1 very neatly articulates the various related agendas of relevance, but the paper itself then fails to articulate the opportunities (or challenges) or integrating a pre-conception agenda with these other agendas of relevance at the same period of the lifecourse.

For example, the authors refer to the challenges faced by South Asia and SubSaharan Africa about reaching MDGs 4 and 5. These regions are particularly challenged by child pregnancy, interpersonal violence in the context of limited gender roles. In these regions, the pre-conception agenda would overlap very well with an adolescent health agenda. In contrast, in high income countries where the age of marriage is much later, this framing may not be as effective. Articulating these types of opportunities/tensions would make the paper feel more academically robust.
In this section, the argument moves from valuing public health programmes across the lifecourse to the recognition that prenatal programmes are weak or nonexistent in LMIC. The authors then suggests that 'in all high income countries prenatal programs are in place to promote good health, prevent health problems and respond to them if they occur during pregnancy'. This is highly debatable. Certainly, where these programs do exist they are not generally viewed as prenatal programs. The reference for this also needs strengthening.

The authors argue for benefits of extending traditional MCH activities to pre-conception care. This has some salience in terms of levering the infrastructure and political goodwill that supports the existing MCH agenda, especially in relation to MDGs 4 and 5. Yet, most of the potential interventions outlined in Box 1 lie well beyond the scope of existing MCH expertise. The argument would be strengthen by more explicit acknowledgement of such a limitation, together with the obvious fact that MCH programs overwhelmingly focus on women. Argument needs to be provided about how such programmes could be expected to prioritize young men's health.

The evidence

As a reviewer, I am not in a position to understand how well the evidence has been elucidated within the previous papers. As mentioned earlier, I have reviewed but one of these and was less impressed with the strength of the evidence presented than the authors in making their 'call to arms'. While there is no doubt about the contribution of the health issues of mothers (and fathers) to poor child and maternal health outcome, and the potential benefits of particular interventions (eg smoking cessation, reduction of too rapid pregnancies), the authors correctly refer to the limited evidence about delivering these interventions in LMIC.
This section would be stronger were there a clearer agenda articulated about the type of research that is now required for this agenda to be advanced.

Potential benefits of preconception care

This section feels as if it is repeating earlier sections about the rationale, albeit with a stronger focus on why addressing men matters. Again, it feels like the same argument that others would make about the importance of mental health and of adolescent health, but using a different lens. And again, the pre-conception argument would be strengthened by more explicit recognition of the overlapping aspects of these agendas, with questioning about how best these different agendas could be articulated, integrated and implemented.

What interventions to deliver, how to deliver them and whom to target.

This section is helpful in articulating how some of these different aspects might be combined, and at a very big picture level, at identifying how countries might prioritise particular interventions. However, to this reviewer, greater ‘grunt’ around how this might happen is required for a paper like this.

The last paragraph of this section (starting with ‘A logical target group…’) would benefit from a diagram to assist the reader to understand these different populations. This section feels as if it would fit better closer to the start of the paper, potentially within the section on ‘what is pre-conception care’. It would also benefit from more consistent articulation of the opportunities and challenges of targeting each of these populations.

Potential risks of preconception care.

This is an important section as there are indeed risks of promulgating a pre-conception agenda as the authors describe. It would be helpful were the authors to provide more detail about how to safeguard implementations efforts to ensure that the risks they describe are obviated. For example, I am aware that in WHO’s SEARO region, discussions about the preconception agenda have suggested a focus on the language of ‘promoting healthy transitions’ for older children and adolescents as a strategy to ensure that both boys and girls are engaged.

Challenges of delivering preconception care in LMIC

The somewhat overblown language in the opening section feels more appropriate for a presentation than an academic paper. The argument would come across more strongly with more balanced language, especially given the substantial challenges of implementation.

The second paragraph suggests that, notwithstanding the scope of potential interventions in Box 1) that the authors are simply wishing to extend the existing MCH agenda. There are clearly some risks in this approach as well as some benefits. The paper would be stronger were this approach to be argued as but one of the potential approaches that could see this agenda implemented – with articulation required of both the opportunities/strengths as well as the challenges/limitations that might come from using a range of programs and platforms.

What will it cost?

The benefits of diabetes interventions is that women with diabetes are typically already linked into the health system. While a nice example with strong health economic support for interventions, it would be beneficial were the authors to comment on the different infrastructure required to deliver more population focused interventions (eg school settings to deliver comprehensive sexuality education).

Minor comments

Reference 5 and 9 do not appear to be available through usual search engines and should be reconsidered.

In Box 1, under Mental Health, the first item is ‘assessing psychosocial problems’. This point could similarly be made for each of the themes; there is no reason why it should only be here. Suggest either omit or add to all. I was looking for the term ‘treatment’ and found it under the line ‘counselling, treating and managing depression’. Using the distinct terms of counselling and treating suggests may suggest that treatment only constitute medication. Unless this is standard WHO speak, I would suggest using the term ‘treat’ which the authors may wish to add including access to counselling, medication and community supports) or some such.

Has Figure 1 been newly developed for this publication or modified from an existing publication. If modified, need to cite a reference (or seek permission).

Referee's comments to the authors– this sheet WILL be seen by the author(s) and published with the article

Title	Preconception care: Advancing from 'important to do and can be done' to 'is being done and is making a difference'
Author(s)	Elizabeth Mason*, Venkatraman Chandra-Mouli, Valentina Baltag, Charlotte Christiansen, Zohra S Lassi, Zulfiqar A Bhutta
Referee's name	Rehana Salam

When assessing the work, please consider the following points, where applicable:

1. Is the question posed by the authors new and well defined?
2. Are the methods appropriate and well described, and are sufficient details provided to replicate the work?
3. Are the data sound and well controlled?
4. Does the manuscript adhere to the relevant standards for reporting and data deposition?
5. Are the discussion and conclusions well balanced and adequately supported by the data?
6. Do the title and abstract accurately convey what has been found?
7. Is the writing acceptable?

Please make your report as constructive and detailed as possible in your comments so that authors have the opportunity to overcome any serious deficiencies that you find and please also divide your comments into the following categories:

General comments: I understand that the paper is the last one in the series, however it should provide some references (as and where applicable) within the text so that the reader can refer to the specific paper for more details.

- Major Compulsory Revisions (which the author must respond to before a decision on publication can be reached)

- Minor Essential Revisions (such as missing labels on figures, or the wrong use of a term, which the author can be trusted to correct)

Abstract looks a bit lengthy. Please check the word count.

Please mention objective/question that the paper aims to address

The objective/ research question not defined.

Authors have tried to consolidate the evidence and recommend plan for action in this final paper of the series. However, it misses linkages with the evidences. Please link to the previous papers earlier in the text to give a perspective to the reader.

In box 1, what is meant by "Examples of evidence-based interventions?"

- Discretionary Revisions (which are recommendations for improvement but which the author can choose to ignore)

Since this paper is more of a commentary/call for action, I suggest it would be better if abstract could be turned into 'key messages' that the authors want to convey to the readers. Abstract in the current form does not portray the content.

Where possible please supply references to substantiate your comments.

When referring to the manuscript please provide specific page and paragraph citations where appropriate.

Comments from the Supplement Editor

The abstract does not reflect the findings of the manuscript and goes beyond the results obtained.

The arguments given in the text are insufficiently rigorous from an academic perspective. Please address this issue, giving more support to your recommendations or softening the emphasis of such conclusions and recommendations.
